# A cross-sectional study of parental perspectives on children about COVID-19 and classification using machine learning models

**DOI:** 10.3389/fpubh.2024.1373883

**Published:** 2025-01-15

**Authors:** Fahmida Kousar, Arshiya Sultana, Marwan Ali Albahar, Manoj Shamkuwar, Md Belal Bin Heyat, Mohd Ammar Bin Hayat, Saba Parveen, John Irish G. Lira, Khaleequr Rahman, Abdullah Alammari, Eram Sayeed

**Affiliations:** ^1^Department of Amraze Atfal, A and U Tibbia College & Hospital, Delhi University, New Delhi, India; ^2^Department of Ilmul Qabalat wa Amraze Niswan, National Institute of Unani Medicine, Ministry of AYUSH, Bengaluru, Karnataka, India; ^3^Computer Science Department, Umm Al-Qura University, Mecca, Saudi Arabia; ^4^Department of Panchkarma, A and U Tibbia College & Hospital, Delhi University, New Delhi, India; ^5^CenBRAIN Neurotech Center of Excellence, School of Engineering, Westlake University, Hangzhou, Zhejiang, China; ^6^College of Intelligent Systems Science and Engineering, Harbin Engineering University, Harbin, China; ^7^College of Electronics and Information Engineering, Shenzhen University, Shenzhen, China; ^8^National University Manila, Manila, Philippines; ^9^Dasmarinas Graduate School, De La Salle University, Dasmarinas, Cavite, Philippines; ^10^Department of Ilmul Saidla, National Institute of Unani Medicine, Ministry of AYUSH, Government of India, Bengaluru, Karnataka, India; ^11^Faculty of Education, Curriculums and Teaching Department, Umm Al-Qura University, Makkah, Saudi Arabia; ^12^Triveni Rai Kisan Mahila Mahavidyalaya, D. D. U. Gorakhpur University, Kushinagar, India

**Keywords:** public health, medical machine learning, parents, children health, SARS CoV-2, healthcare, medical intelligence, kid

## Abstract

**Background and objective:**

This study delves into the parenting cognition perspectives on COVID-19 in children, exploring symptoms, transmission modes, and protective measures. It aims to correlate these perspectives with sociodemographic factors and employ advanced machine-learning techniques for comprehensive analysis.

**Method:**

Data collection involved a semi-structured questionnaire covering parental knowledge and attitude on COVID-19 symptoms, transmission, protective measures, and government satisfaction. The analysis utilised the Generalised Linear Regression Model (GLM), K-Nearest Neighbours (KNN), Support Vector Machine (SVM), Random Forest (RF), Naive Bayes (NB), and AdaBoost (AB).

**Results:**

The study revealed an average knowledge score of 18.02 ± 2.9, with 43.2 and 52.9% of parents demonstrating excellent and good knowledge, respectively. News channels (85%) emerged as the primary information source. Commonly reported symptoms included cough (96.47%) and fever (95.6%). GLM analysis indicated lower awareness in rural areas (*β* = −0.137, *p* < 0.001), lower attitude scores in males compared to females (*β* = −0.64, *p* = 0.025), and a correlation between lower socioeconomic status and attitude scores (*β* = −0.048, *p* = 0.009). The SVM classifier achieved the highest performance (66.70%) in classification tasks.

**Conclusion:**

This study offers valuable insights into parental attitudes towards COVID-19 in children, highlighting symptom recognition, transmission awareness, and preventive practices. Correlating these insights with sociodemographic factors underscores the need for tailored educational initiatives, particularly in rural areas, and for addressing gender and socioeconomic disparities. The efficacy of advanced analytics, exemplified by the SVM classifier, underscores the potential for informed decision-making in public health communication and targeted interventions, ultimately empowering parents to safeguard their children’s well-being amidst the ongoing pandemic.

## 1 Introduction

Coronavirus disease is exclusive and unique in several aspects, and the COVID-19 pandemic has challenged healthcare systems (1). The World Health Organisation (WHO) declared the situation a public health emergency of international concern (2, 3). In 2021, a significant resurgence of infections occurred worldwide, prompting parents in India to seek guidance on measures to protect their children from this highly contagious virus, particularly when symptoms were present or a positive test result had been received (4). As of September 21, 2021, India reported over 33.5 million confirmed cases of SARS-CoV-2, with a recovery rate of 97.5% and a mortality rate of 1.33%. During the second wave, the pandemic’s impact on children raised alarm among parents, highlighting the urgent need for measures to support children worldwide and prompting warnings from healthcare organisations in India (accessed on 20 June 2023).^1^ During the second wave of the COVID-19 pandemic, the effects on children raised significant concerns among parents. The broader implications for children’s health highlighted the urgent need for action to support them globally, along with warnings from healthcare agencies in India (4, 5). However, it remains uncertain whether children exhibit greater resistance to certain infectious diseases due to their robust innate immunity and healthier respiratory systems compared to adults, as well as the potential presence of fewer underlying health issues in children (6). Reports indicate that children under 12 years old in India were infected with COVID-19, which contrasts with the common belief that the virus primarily spares this age group and that only a small number of children are affected (4). Additionally, the lower number of COVID-19 infections in children during the second wave may be attributed to reduced international travel and limited participation in outdoor activities, which likely decreased their exposure to the virus (6). During the second wave of COVID-19 since March, an intensive care paediatrician reported that at Delhi’s Gangaram Hospital, India, more than 75 patients were seen, aged between 4 and 15 years (accessed on 2 May 2022).^2^

Children have been significantly affected by the COVID-19 pandemic, facing social isolation, disrupted routines, increased screen time, and limited physical activities, which contributed to mental health issues such as anxiety and depression. Excessive screen time has been associated with negative impacts on cognitive and socio-emotional development, as well as sleep disruption, further affecting physical and psychological health (7–9). Children typically possess fewer personal resources than adults to manage sudden changes, which often leads them to rely on their parents for support, particularly when they are unable to engage with other adults, such as teachers and grandparents. In the absence of substantial support from family, friends, or community organisations, heightened parental stress may adversely affect children’s physical and psychological well-being, resulting in alterations to parenting behaviours (10, 11). Studies have shown that parental stress is a predictor of child abuse (12). The mental health of children is intricately connected to that of their parents, whereby residing with a parent experiencing poor mental health significantly elevates the likelihood of the child experiencing similar challenges with their mental well-being (13). The heightened stress and additional responsibilities parents experienced during the pandemic may have led to a diminished level of adult support available to children in their daily lives. This insufficient support can impede their neurobiological and socio-emotional development (14). During significant public health crises and social unrest, individual perceptions of risk can profoundly influence emotional responses, behaviours, and decision-making processes. Existing research highlights a positive correlation between risk perception and the experience of negative emotions. In this context, developing and applying effective parenting skills become crucial, particularly when children are confined at home. These skills not only safeguard the well-being of children but also contribute to the broader goal of protecting the community from the impacts of COVID-19 (15–17).

Three categories of child adversities were noted, i.e., (i) Children COVID-positive and isolated, (ii) Children who have lost one or both parents due to the COVID-19 infection and whose parents are tested positive for COVID-19, and (iii) children due to lockdown, who are in quarantine or isolated. These children may face different sets of mental health issues related to different categories (4).

Multisystem inflammatory syndrome in children (MIS-C) emerges as a severe paediatric complication linked to COVID-19. MIS-C presents as an inflammatory syndrome capable of impacting nearly every organ system (18, 19). The diverse set of clinical conditions of MIS-C in children includes fever, coagulopathy, hypotension, shock, cardiac involvement, acute gastrointestinal symptoms as well as respiratory, renal, dermatologic, or neurologic ailments. Although fever and gastrointestinal symptoms rank as the most prevalent, neurologic, and dermatologic manifestations are also thoroughly documented. Diagnosis involves a combination of clinical and laboratory testing, with patients often exhibiting elevated inflammatory markers elevated CRP, ESR, fibrinogen, D-dimer, IL-6, and elevated neutrophils. Further, potential abnormalities can be observed in electrocardiograms or echocardiograms (18). Hence parents should have knowledge of COVID-19 symptoms in children.

Conventional medicine is crucial in addressing epidemic threats, especially in critical care situations. However, recent preventive measures have proven insufficient, and alternative options are still being explored. During this global crisis, the preventive and therapeutic potential of traditional and complementary medicine is not being fully considered (1). However, it is a crucial time to embrace integration with integrative approaches to explore the solutions to the COVID-19 catastrophe. In India, very scarce data-based studies have been conducted. In-depth surveys and clinical studies are essential to understand the epidemiology of COVID-19 during the pandemic (4). Hence, a research study is required to determine the knowledge and attitude of parents towards the symptoms of COVID-19 among children, modes of transmission, and protective measures. Primarily, traditional healthcare is useful for the prevention and treatment of illness (20).

Previously, Person et al. (21) found that during disease outbreaks, targeted health education materials contribute to extenuating fear, stigmatisation, and discernment (22). The broad disease spectrum, the early impression that paediatric infections were infrequent and usually mild has been substituted by a more thoughtfulness of infectious appearance in children across countries (23). Indian citizens’ knowledge, attitudes, practices, and awareness affect their adherence to COVID-19 control measures (2). It is crucial to focus on raising awareness, especially among children, as an essential strategy for reducing COVID-19 transmission. Therefore, assessing parents’ knowledge and attitudes about COVID-19 infection is of paramount importance. While some research has been conducted in this area (22, 24) there is still a gap in understanding parents’ knowledge and attitudes concerning COVID-19 symptoms in their children, modes of transmission, protective measures, and their awareness of home remedies for prevention or treatment of COVID-19 symptoms, as highlighted in a previous study (25). The knowledge and attitude of parents on symptoms of COVID-19 in their children, mode of transmission, protection measures, and their knowledge about the use of home remedies to prevent or treat COVID-19 symptoms has yet not been studied (17).

This research goals to fill the gap by examining parents’ knowledge and attitudes regarding symptoms of COVID-19 infection among children, transmission, and protective measures. We used machine learning-based models such as k-Nearest Neighbour (KNN), AdaBoost (AB), Random Forest (RF), and Naive Bayes (NB) to analyse the collected data. Machine learning excels at recognising complex patterns in large datasets, making it ideal for uncovering subtle correlations in the multifaceted nature of parental perspectives. Moreover, machine learning models, such as decision trees and neural networks, are adept at capturing non-linear relationships. This is crucial when studying human behaviour, especially attitudes, which often involve intricate, non-linear associations that traditional statistical methods may struggle to grasp. The predictive analysis capability of machine learning is valuable for forecasting potential shifts in parental attitudes and identifying knowledge gaps. By training models to predict outcomes based on given features, your study can take a proactive approach to addressing emerging concerns related to COVID-19. Machine learning also facilitates the identification of feature importance (26–28), helping pinpoint which factors significantly contribute to parental knowledge and attitudes. This analysis can inform interventions and educational campaigns by targeting the most significant aspects and optimising resource allocation. Given the ever-changing nature of the COVID-19 situation, the adaptability of machine learning is a major advantage. Models can be retrained with new data, enabling the study to evolve in response to the shifting landscape of information and public sentiment. The main contributions of this study are as follows:

- This research presented insights into parents’ knowledge and attitudes regarding COVID-19 infection symptoms among children, the mode of transmission, and protective measures.
- This study established correlations between sociodemographic parameters and parents’ knowledge and attitudes regarding COVID-19 among children.
- We used machine learning models to classify COVID-19-related modes of transmission, and prevention, as well as parents’ knowledge and attitudes.

## 2 Materials and methods

**Figure 1:**
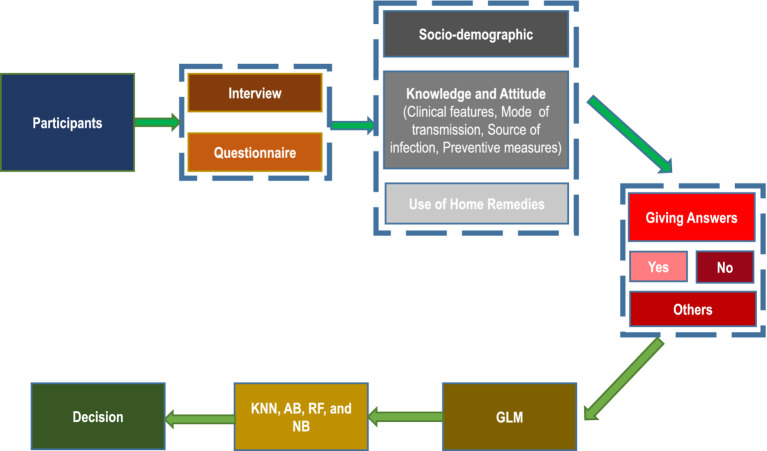
Organisational diagram of the proposed study.

A hospital-based observational cross-sectional study was conducted with 340 parents, as illustrated in Figure 1. The research took place in the outpatient Department of Paediatrics at A & U Tibbia College in New Delhi, India, from December 2021 to April 2022. This district-level medical college features a 300-bed hospital. In this study, “parents” refers to parents or guardians. Parents visiting the Paediatrics outpatient department with their children were surveyed about their knowledge of COVID-19 symptoms, regardless of whether their children tested positive for the virus, exhibited COVID-19-like symptoms, or neither.

### 2.1 Scientific review and ethical consideration

This intramural research protocol was approved by the Scientific and Ethical Committee (F.5 (283)/2013-CO/1620, dated 14/12/2021) of the Institute. This study followed the Helsinki Declaration and the GCP guidelines, Ministry of AYUSH, Government of India. The study was registered in the CTRI (CTRI/2021/12/038788, dated: 21/12/2021). Informed consent was obtained from all the participants who were involved in the study.

### 2.2 Experimental data collection

Parents of children who satisfied the inclusion criteria were given a detailed description of the study in the information sheet regarding the nature of the study and adequate time to go through it. They were allowed to ask any question regarding the study, and if they agreed to participate in the study, they were marked as agreed by informed consent in the Google e-form.

Parents of any gender with children under 18 years’ old who were willing to participate in the study were included, while those unwilling to participate were excluded. Educated parents proficient in English completed the e-Google form directly. For illiterate parents, the questionnaire was translated into the local language and filled out by the investigator. The semi-structured questionnaire gathered demographic data and assessed parents’ knowledge and attitudes regarding COVID-19 symptoms, modes of transmission, protective measures, sources of information, and their satisfaction with government actions related to COVID-19 for children. Additionally, the questionnaire explored parents’ knowledge of home remedies that could help prevent and treat COVID-19 symptoms.

The data were collected through an e-form questionnaire, with the first section covering general information such as age, gender, religion, socioeconomic status (assessed using Kuppuswamy’s socioeconomic scale, 2018), habitat, and sources of information extracted from the e-case record form. Kuppuswamy’s socioeconomic scale, developed in 1976, combines the education and occupation of the family head with the family’s monthly income to yield a composite score ranging from 3 to 29. The scale categorises families into five groups: “upper class, upper-middle class, lower-middle class, upper-lower class, and lower socioeconomic class” (29, 30). The second section included a pre-test valid and reliable instrument with Cronbach alpha = 0.85 used by Abu Hammad (17). This instrument included the knowledge and attitude of parents regarding COVID-19 infection among children. It consists of 24 points: 11 points for clinical symptoms, four points for modes of transmission of COVID-19, nine points for protective measures against COVID-19 to protect children, and two points for satisfaction with governmental measures. Each response was given zero points if it was wrong and one point for the right (17). The knowledge scores were categorised based on the total scores for each responder. A score between 17 and 24 was considered excellent, 9 and 16 were considered good, and 0 and 8 were considered poor knowledge and attitude.

### 2.3 Sample size

The sample size for this study was determined using computer-based statistical software. Given a prevalence rate of 70% of children with COVID-19 being asymptomatic, a confidence level of 95% was established, along with a total margin of error set at 10%. Consequently, the required sample size was calculated to be 329 participants; however, our study included 340 participants. The duration of the study spanned 4 months, commencing on December 22, 2021, and concluding on April 25, 2022. Participants were enrolled per the Clinical Trials Registry of India (CTRI). Parents or guardians attending the Paediatric Outpatient Department (OPD) with their children and expressing a willingness to participate were included in the study. A systematic random sampling technique was employed, where every third parent entering the OPD was approached for an interview.

### 2.4 Statistical methods

The data were imputed in Microsoft Excel and analysed by the statistical software SPSS 28 version for Windows (IBM Corporation, Armonk, NY). Results of quantitative variables were described by mean ± S.D., and qualitative variables were described by frequency (%). Appropriate correlation coefficient tests were used to find an association between COVID-19 symptoms and sociodemographic factors. The knowledge score was categorised after calculating the total scores for each responder. A generalised linear regression model (GLM) was used to find the factors affecting the overall knowledge, attitude, and belief scores, as the Gamma distribution using the log link function was imputed since the error distribution was skewed. All *p*-values were two-tailed, and an alpha error was set as 0.05.

### 2.5 Machine learning techniques

In this study, we utilised various machine learning classifiers to classify modes of transmission, prevention strategies, as well as parents’ knowledge and attitudes. Among the classifiers employed were K-Nearest Neighbours (KNN), Support Vector Machine (SVM), Random Forest (RF), Naive Bayes (NB), and AdaBoost (AB). In conjunction with these classifiers, we employed cross-validation (CV) techniques, including 2- and 3-fold CV, random sampling, and leave-one-out models. The detailed descriptions of the classifiers are as follows:

KNN is a non-parametric classification algorithm that assigns a class label to a data point based on the majority class of its k nearest neighbours in the feature space (27). Mathematically, for a given data point *x*, the predicted class *y* can be expressed as Equation 1:


(1) $$y=\mathrm{majority vote}\;\left(y_{1},y_{2},\ldots ,y_{k}\right)\text{.}$$

where *y*_1_, *y*_2_,…,*y_k_* are the class labels of the k nearest neighbours of *x*.

SVM is a supervised machine learning algorithm used for classification tasks. It is used to find the hyperplane that best separates the classes in the feature space (31). Mathematically, for linearly separable data, the decision boundary can be represented in Equation 2:


(2) $$w^{T}\;x+b=0.$$

where *w* is the weight vector, *x* is the input vector, and *b* is the bias term.

NB is a probabilistic classifier based on Bayes’ theorem with the assumption of independence between features (32). Mathematically, the probability of a class given the features can be expressed in Equation 3:


(3) $$P\left(\frac{y}{x_{1,\;\;\;x_{2},\ldots ,x_{n}}}\right)=\frac{P\left(y\right)*P\left(\frac{x_{1}}{y}\right)*P\left(\frac{x_{2}}{y}\right)*\ldots ..*P\left(\frac{x_{n}}{y}\right)}{P\left(x_{1}\right)*P\left(x_{1}\right)*\ldots \ldots .*P\left(x_{n}\right)}$$

AB is an ensemble learning method that combines multiple weak classifiers to form a strong classifier (33). Mathematically, the final classification can be represented as a weighted sum of the individual weak classifiers as mentioned in Equation 4:


(4) $$F\left(x\right)=\sum_{t-1}^{T}\mathrm{\alpha tft}\left(x\right)$$

where *F* (*x*) is the final classification, *f_t_*(*x*)is the t-th weak classifier, and *α_t_* is the weight assigned to the t-th weak classifier.

### 2.6 Performance evaluation methods

We used KNN, SVM, RF, NB, and AB classifiers with 2- and 3-fold cross-validation (CV), random sampling, and leave-one-out models to classify COVID-19-related modes of transmission, prevention, knowledge, and attitudes. Additionally, performance measures such as AUC, accuracy, F1, precision, recall, and specificity were calculated (34). The standard mathematical expressions of the accuracy, F1, precision, recall, and specificity are mentioned in the Equations 5–9 (35–37):


(5) $$\begin{array}{l} \mathrm{Accuracy}\;\left(\%\right)=\\ \frac{\mathrm{True Postive}+\mathrm{True Negative}}{\mathrm{False Postive}+\mathrm{False Negative}+\mathrm{True Negative}+\mathrm{True Positive}}\times 100 \end{array}$$

(6) $$F1\;\left(\%\right)=\frac{2\times \mathrm{Recall}\times \mathrm{Precision}}{\mathrm{Recall}+\mathrm{Precision}}\times 100$$

(7) $$\mathrm{Precision}\;\left(\%\right)=\frac{\mathrm{True Positive}}{\mathrm{True Positive}+\mathrm{False Positive}}\times 100$$

(8) $$\mathrm{Recall}\;\left(\%\right)=\frac{\mathrm{True Positive}}{\mathrm{True Positive}+\mathrm{False Negative}}\times 100$$

(9) $$\mathrm{Specificity}\;\left(\%\right)=\frac{\mathrm{True Negative}}{\mathrm{True Negative}+\mathrm{False Positive}}\times 100$$

## 3 Results

**Figure 2:**
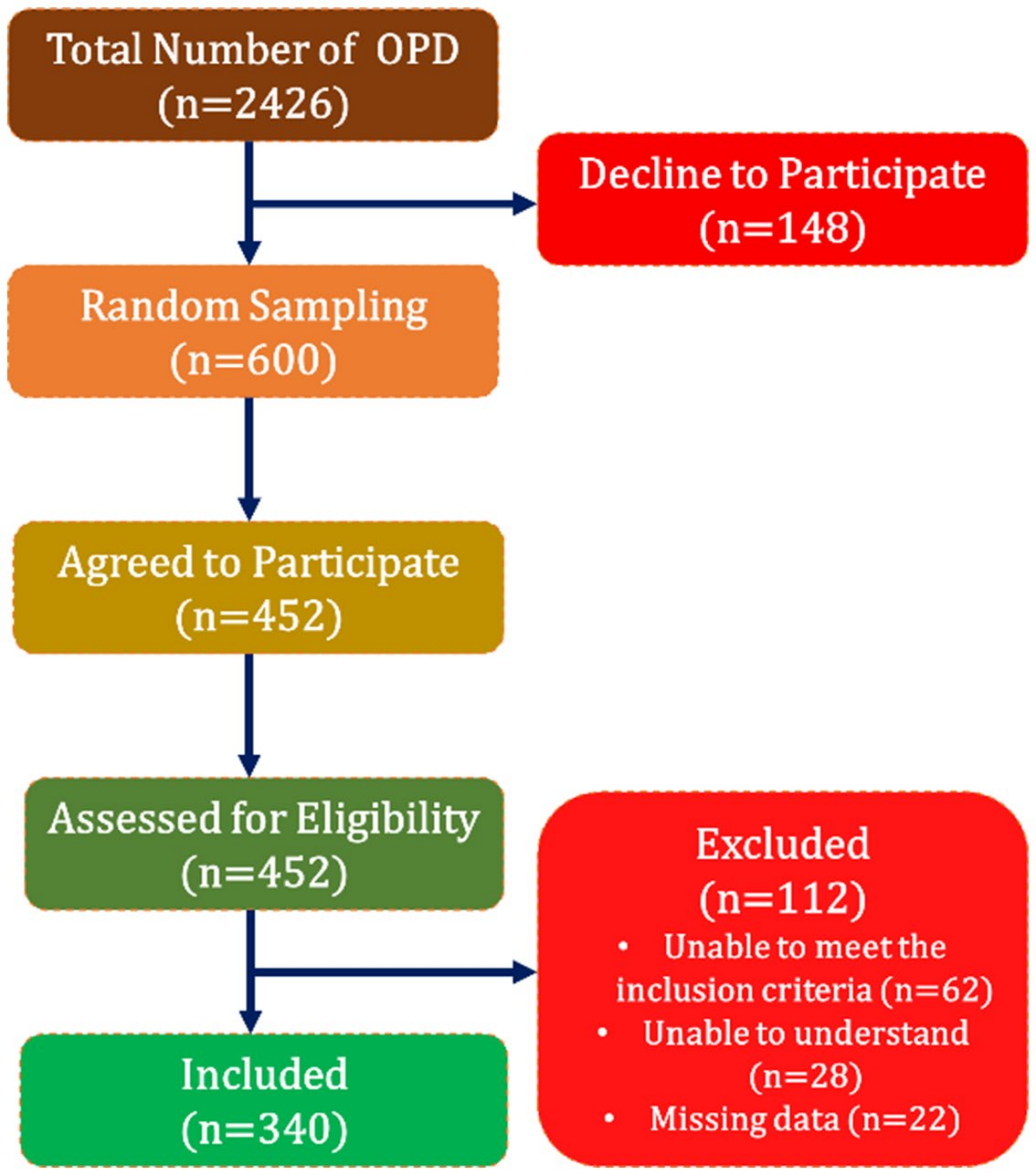
Flowchart of the participants.

Out of the 2,426 children accompanied by their parent/guardian who attended the outpatient department (OPD), 600 were screened for participation. Following systematic random sampling, every third parent was invited to participate in the study. However, 148 parents declined to participate. Among the parents who consented, eligibility assessments excluded 112 parents, resulting in the inclusion of 340 parents in the study (Figure 2).

The average age of the parents was 35.08 ± 8.08 years. In this study, the majority of the parents fell within the 29–38 age range, comprising 47.64% of the sample (*n* = 162). The study observed that most of the parents were females, accounting for 65.29% of the total (*n* = 222/340) and resided in urban areas (92.1%; *n* = 313). Regarding socioeconomic status, the findings indicated that a significant portion of parents had completed education up to the middle school certificate level (27.35%; *n* = 93), engaged in elementary occupations (67.75%; *n* = 230), and reported a family income ranging between Rs 6,175–18,496 (77.06%; *n* = 262). The socioeconomic status was predominantly categorised as upper lower (72.06%; *n* = 245; Table 1).

**Table 1:** Sociodemographic parameters of the parents.

Variables	Frequency	%	Variables	Frequency	%
**Age group (years)**					
≤18	4	1.18	Technicians and Associate professionals (8)	4	1.20
19–28	70	20.59	Professionals (9)	10	2.90
29–38	162	47.65	**Education of the head of the family**		
39–48	85	25.00	Illiterate (1)	80	23.50
49–58	14	4.12	Primary school certificate (2)	22	6.50
59 and above	5	1.47	Middle school certificate (3)	93	27.40
**Gender**			High school certificate (4)	87	25.60
Female	222	65.29	Intermediate or diploma (5)	22	6.50
Male	118	34.71	Graduate (6)	33	9.70
**Religion**			Profession or Honours (7)	3	0.90
Muslims	252	74.10	**Total monthly income of the family**		
Hindu	87	25.60	< 6,326 (1)	19	5.60
Other	1	0.30	6,327–18,952 (2)	262	77.05
**Habitat**			18,953–31,590 (3)	40	11.76
Rural	27	7.90	31,591–47,265(4)	11	3.20
Urban	313	92.10	47,266–63,181(6)	6	1.80
**Socioeconomic status as per Kuppuswamy SES scale**			63,182–126,359(10)	2	0.60
**Occupation of the head of the family**			≥ 126,360	0	0
Unemployed (1)	4	1.20	**Socioeconomic status (SES) score**		
Elementary Occupation (2)	230	67.60	Lower (V): <5	8	2.40
Plant & Machine Operators and Assemblers (3)	7	2.10	Upper lower (IV):5–10	245	72.10
Craft & Related Trade Workers (4)	14	4.10	Lower middle (III):11–15	71	20.90
skilled agriculture or fishery worker (5)	5	1.50	Upper Middle (II)16–25	15	4.40
Skilled workers and shop & Market Sales (6)	60	17.60	Upper (I):26–29	1	0.30
Clerks (7)	6	1.80	Total	340	100.00

### 3.1 Knowledge and attitudes of parents regarding COVID-19 symptoms, major source of information, mode of transmission, and preventive measures against COVID-19

Parents indicated that the most commonly observed initial clinical symptoms of COVID-19 were cough (96.5%, *n* = 328) and fever (95.6%, *n* = 325). Additionally, 31.2% of parents believed that children could be carriers of the virus without displaying symptoms. The primary channels through which parents gathered information about COVID-19 were news channels (85%, *n* = 289/340), as well as input from family, friends, and neighbours (82.1%, *n* = 279/340) and other sources accounting for 35.3% (*n* = 120/340). All surveyed parents agreed that the transmission of COVID-19 among children involves multiple factors and modes. In this study, parents identified droplet infection as the primary mode of transmission (93.2%, *n* = 317). Table 2 provides a summary of the protective and governmental measures recommended to prevent the spread of COVID-19.

**Table 2:** Responses to knowledge and attitude of parents for COVID-19 infection among children.

Knowledge regarding symptoms	Responses (Yes)	%	Responses (No)	%
Fever	325	95.59	15	4.41
Cough	328	96.47	12	3.53
Running nose	306	90.00	34	10.00
Sore throat	310	91.18	30	8.82
Shortness of breath	301	88.53	39	11.47
Joint muscle pain	142	41.76	198	58.24
Red eyes	85	25.00	255	75.00
Skin rash	53	15.59	287	84.41
Diarrhoea	43	12.65	297	87.35
Vomiting	62	18.24	278	81.76
Asymptomatic	106	31.18	234	68.82
Mode of transmission				
Shaking hands	316	92.94	24	7.06
Contacting Surface	298	87.65	42	12.35
Asymptomatic Person	158	46.47	182	53.53
Respiratory Droplet	317	93.24	23	6.76
Preventive measure				
Frequently Washing hand	335	98.53	5	1.47
Eat boiled and cooked food	321	94.41	19	5.59
Apply Mask	338	99.41	2	0.59
Apply mask to suspected patients	331	97.35	9	2.65
The suspected patient is in a well-ventilated individual room	330	97.06	10	2.94
Health workers must wear protective clothing	331	97.35	9	2.65
Avoid transporting patients and transporting them outside their area unless necessary	331	97.35	9	2.65
Routinely cleaning and disinfecting surfaces in connection with suspected patients	332	97.65	8	2.35
Satisfaction with Governmental measures against COVID-19	Number	%		
Parents supported the government initiatives to curb COVID-19	330	97.10		
Quarantining all members of the family	330	97.10		
Educating individuals on COVID-19	332	97.60		
Closing of the School	322	94.70		

### 3.2 Knowledge and attitude score of parents

The mean knowledge score was 18.02 ± 2.91. In the overall assessment, 52.9% (*n* = 180) of parents demonstrated good knowledge, while 43.2% (*n* = 147) exhibited excellent knowledge about COVID-19 infection. Conversely, a small proportion, 3.8%, of parents had poor knowledge of the subject (Table 3).

**Table 3:** Multivariable GLM for parents’ knowledge, attitudes, and beliefs in COVID-19 infection among children.

Demographic variables	Overall		Knowledge		Attitudes		Belief	
	β (95%CI)	*p*-value	β (95%CI)	*p*-value	β (95%CI)	*p*-value	β (95%CI)	*p*-value
Gender								
Female	ref		ref		ref		ref	
Male	0.05(−0.021, 0.032)	0.685	0.015(−0.021, 0.051)	0.413	−0.64(−0.120, −0.008)	0.025*	−0.047(−0.077, −0.017)	0.002*
Education								
Literate	ref		ref		ref		ref	
Illiterate	−0.025(−0.55, 0.004)	0.093	−0.037(−0.077, 0.003)	0.073	0.006(−0.30, 0.041)	0.755	−0.003(−0.037, 0.031)	0.875
Habitation								
Urban	ref		ref		ref		ref	
Rural	−0.137(−0.185, −0.091)	<0.001*	−0.168(−0.231, −0.106)	<0.001*	−0.064(−0.120, 0.044)	0.481	−0.069(−0.122, −0.016)	0.011*
Socioeconomic status (SES)								
Upper	ref		ref		ref		ref	
lower	0.001(−0.028, 0.031)	0.927	0.015(−0.030, 0.051)	0.611	−0.048(−0.083—0.021)	0.009*	0.007(−0.027, 0.040)	0.707
Age (years)								
≤50	ref		ref		ref		ref	
>50	0.023(−0.037, 0.082)	0.455	0.037(−0.044, 0.118)	0.370	0.005(−0.067, 0.078)	0.888	−0.027(−0.096, 0.041)	0.433

β (95% Wald CI) was derived from a GLM (Gamma distribution with log link function); *p* < 0.01, high significant difference; *p* < 0.001, extremely significant difference; Ref, Reference.

### 3.3 Association between knowledge, attitude, and belief with sociodemographic variables

Table 3 presents the results of a multivariable Generalised Linear Model (GLM) analysis examining the association between parents’ knowledge, attitudes, and beliefs regarding COVID-19 infection among children and various sociodemographic variables. The analysis revealed that habitation significantly influenced knowledge and attitude scores. Specifically, individuals in rural areas demonstrated lower awareness compared to their urban counterparts, as indicated by a beta coefficient of −0.137. This suggests an inverse relationship between awareness scores in rural areas and urban areas, with statistical significance (*p* < 0.001). Moreover, attitudes were found to be influenced by gender and socioeconomic status. Males exhibited a decrease in attitude scores compared to females, with a rate of change in the beta coefficient of −0.64 and a *p*-value of 0.025. Individuals with lower socioeconomic status experienced a decline in attitude scores compared to those with upper socioeconomic status, with a rate of change in the beta coefficient of −0.048 and a *p*-value of 0.009. Similarly, gender and habitat emerged as factors associated with the belief domain and statistical significance of (*p* < 0.05).

### 3.4 Classification results

In this study, various classifiers such as KNN, SVM, RF, NB, and AB were employed and used in conjunction with 2- and 3-fold cross-validation (CV), random sampling, and leave-one-out models to classify COVID-19-related modes of transmission, prevention, parents’ knowledge and attitudes. Performance metrics such as AUC, accuracy, F1 score, precision, recall, and specificity were computed for analysis. Table 4 displays the findings obtained and this revealed that SVM classifiers achieved the highest accuracy (66.70%) with random sampling, while AB classifiers demonstrated the lowest accuracy (16.70%). Prior research by Vabalas et al. (38) indicated that CV models alone may not adequately address overfitting concerns. Similarly, Varoquaux et al. (39) suggested that CV models do not necessarily offer superior accuracy compared to other methods. However, considering the limited sample size of the study, random sampling and leave-one-out CV models were opted for.

**Table 4:** Classification results based on machine learning models.

Classifier	Model	AUC	Accuracy	F1	Precision	Recall	Specificity
KNN	CV-2	66.20	51.20	49.60	52.50	51.20	63.60
**SVM**		49.30	**63.40**	49.20	40.20	63.40	36.60
RF		55.90	46.30	44.90	43.70	46.30	45.70
NB		58.10	31.70	17.20	11.80	31.70	83.30
AB		37.80	36.60	35.30	34.10	36.60	32.70
KNN	CV-3	61.60	53.70	51.70	55.60	53.70	67.80
**SVM**		44.80	**63.40**	49.20	40.20	63.40	36.60
RF		45.60	39.00	39.50	39.90	39.00	44.70
NB		52.10	29.30	19.20	73.40	29.30	82.60
AB		35.60	39.00	39.50	39.90	39.00	44.70
KNN	Random Sampling (training size: 95%)	43.60	33.30	32.70	53.30	33.30	68.30
**SVM**		62.70	**66.70**	53.30	44.40	66.70	33.30
RF		45.80	33.30	37.90	43.90	33.30	43.30
NB		60.60	33.30	20.20	14.50	33.30	78.30
AB		27.50	16.70	19.80	25.30	16.70	30.00
KNN	Leave-one-out	45.90	36.60	33.40	62.40	36.60	78.80
**SVM**		25.60	**63.40**	49.20	40.20	63.40	36.60
RF		43.00	43.90	40.10	36.80	43.90	39.00
NB		59.40	29.30	15.20	10.30	29.30	81.20
AB		36.40	29.30	29.80	30.40	29.30	34.70

Bold values means highest accuracy.

## 4 Discussion

### 4.1 Major findings and comparison with previous studies

This investigation examines parents’ understanding and perceptions regarding COVID-19 symptoms in children, methods of transmission, and available protective measures. The findings indicate that most parents surveyed fall within the age range of 29 to 38 years (47.65%), with a notable predominance of female respondents (65.27%). The study also highlighted that most children were accompanied by their mothers (58.5%). A significant proportion of parents resided in urban areas (92.1%) and belonged to the upper-lower socioeconomic status (72.06%). Upon analysis using GLM, it was observed that habitation significantly influenced knowledge and attitude. Rural areas exhibited lower awareness compared to urban areas in India. Findings from this study further indicated that attitudes were influenced by factors such as gender and socioeconomic status, with males showing a decrease in attitude scores compared to females and lower socioeconomic status correlating with decreased attitude scores compared to upper socioeconomic status. Similarly, gender and habitat were identified as factors associated with the belief domain. Prakash et al. (2) reported significant variations in attitudes towards controlling COVID-19 based on gender, occupation categories, education levels, and residence. It is noteworthy that parents demonstrated awareness of the COVID-19 pandemic regardless of their education status and family income, aligning with similar findings (40). This study reveals that a significant majority of parents, accounting for 85%, primarily rely on news channels as their source of information (41). Additionally, research conducted by Abu Hammad (18) indicates that the public increasingly depends on social media platforms, including Twitter and Facebook, for news. It is essential, however, for the public to remain vigilant regarding the reliability of information disseminated through these channels and other available resources. Furthermore, the study found that the average knowledge score among parents was 18.02 ± 2.91. In terms of overall knowledge, 52.9% (*n* = 180) and 43.2% (*n* = 147) of parents demonstrated good and excellent knowledge, respectively. These findings are consistent with the study conducted by Abu Hammad (17), where parents also exhibited good knowledge levels.

This study identified parents’ fever (95.6%), cough (96.5%), sore throat (91.2%), and shortness of breath (88.5%) as the most recognised symptoms in children. These findings align with previous research indicating that children and adults share similar symptoms, particularly cough and fever (17, 41–43). Sari et al., also noted that the commonly known symptoms of COVID-19 among children were fever and cough. However, these studies acknowledged that some children exhibited mild symptoms such as skin rash, runny nose, and diarrhoea (17, 41–43). The challenges in diagnosing and managing COVID-19 in children were discussed in these studies, emphasising the difficulty due to the mild or moderate clinical course, and the occurrence of asymptomatic infections (41). In a study conducted during the COVID-19 outbreak in Wuhan and Huangshi City, the Knowledge, Attitude, and Practice (KAP) towards COVID-19 among students in two primary schools in Hubei Province were investigated. Another study found that the clinical course of COVID-19 among children was less severe than that reported in adults. It was also discovered that among 41 children admitted with COVID-19 infection, comorbidities were more prevalent (61%). Panton-Valentine leucocidin *S. aureus* also presents as an initial flu-like prodrome and multi-lobar consolidation accompanied by cavitation (44). Additionally, a study reported that a significant percentage (94.59%) of parents paid considerable attention to COVID-19 and explained it to their children, including those visiting the dental department.

This study found that nearly 93.2% of parents believed that the primary transmission route for COVID-19 was respiratory droplet infection, followed by shaking hands (92.9%). This aligns with the conclusions of other studies (2, 17, 45, 46). The majority of parents indicated that effective preventive measures for COVID-19 included the use of masks for known or suspected patients (99.4%) and frequent handwashing (98.5%). Furthermore, a substantial portion of parents (90%) emphasised that washing hands with water and soap was the most effective way to protect against COVID-19. This is consistent with earlier research suggesting that handwashing with water and soap is a crucial method for breaking the chain of infection. Previous studies highlighted that parents were actively engaged in cleaning and disinfecting surfaces (47–50). The COVID-19 pandemic has not only strained the healthcare system but has also prompted a re-evaluation of the importance of infection prevention through control measures. These measures have the potential to impact the dynamics of various allergic and infectious diseases, positively influencing the burden and spending of the healthcare system during epidemic seasons (51–54). The implemented preventive measures, including social distancing, stay-at-home orders, travel restrictions, facial masking, and continuous sanitization of surfaces, have demonstrated positive effects. These measures have not only reduced direct person-to-person contact but have also contributed to limiting the spread of respiratory viruses and the infiltration of allergens and environmental pollutants, which are significant bronchial irritants (55, 56). Handwashing and the use of disinfectants have been effective against enveloped RNA viruses, particularly sensitive to soaps and detergents, leading to a reduction in the transmission of infectious pathogens (57). However, concerns about the potential adverse effects of face masks on respiratory function, particularly for children, have been raised by some parents, despite a lack of scientific evidence supporting such concerns (53).

This study revealed that over 94.7% of parents supported the decision to close schools. Across the nation, schools were shut down in response to government orders as an immediate measure to control the spread of the first and second waves of COVID-19. The Indian education system, in collaboration with the government, successfully implemented emergency home school programmes through online classes. Additionally, virtual learning was initiated in various regions of the country, providing parents with well-organised access to courses during the lockdown period. These initiatives aimed to alleviate concerns among parents regarding their children’s educational progress, ensuring that education continued without interruption (17). Other studies highlighted stringent containment measures such as the closure of schools, parks, and recreational activities, as well as stay-at-home orders and adverse psychological effects associated with these measures, particularly on children. The disruption of regular routines among these children led to distress, confusion, anxiety, and hostility, potentially impacting their psychosocial well-being and cultural education in the long term (58, 59). Since the onset of the COVID-19 pandemic, regions worldwide have implemented containment measures. As such, this study proposes that local governments and organisations from diverse regions should collaborate to minimise unnecessary barriers and facilitate the rational movement of resources and personnel (60).

### 4.2 Benefits and limitation of home remedies in COVID-19

Additional knowledge regarding home remedies utilised was also noted and a total of 160 (47.05%) parents disclosed that they utilised home remedies/herbs to prevent COVID-19 infection. This study was conducted in India, where the general population was significantly impacted by COVID-19. During the COVID-19 pandemic, many individuals in India, where the pandemic significantly impacted the population, turned to home remedies and herbal treatments to prevent or alleviate symptoms of infection. The use of the AYUSH (Ayurveda, Yoga & Naturopathy, Unani, Siddha, and Homoeopathy) system of medicine is deeply ingrained in the culture, and a majority of the population often relies on traditional herbal remedies for various health issues. This practice extended to COVID-19, with individuals using herbs and home-based solutions to manage mild to moderate symptoms. The Government of India (GOI) also recognised and supported the use of herbal medicines during the pandemic. While no concise, nationwide evaluation has been conducted regarding the efficacy of these remedies in treating COVID-19, numerous herbs and traditional formulations have long been part of India’s medical history. Similarly, during the COVID-19 pandemic, individuals in the general population turned to home remedies and herbs to address mild to moderate symptoms. Consequently, an overview of the utilisation of home/herbal remedies during the COVID-19 period. It is important to note that no concise evaluation or commentary has been conducted regarding the efficacy of the following remedies. Liquorice, winter cherry, turmeric, black pepper, black cumin, ginger, holy basil, moonseed, cinnamon, gooseberry, garlic, and flax seeds possess a long-standing history of use as herbal remedies for various ailments. These herbs are widely utilised in culinary practices and traditional medicine across numerous countries, particularly in India. Additionally, traditional Indian formulations like Triphala and Rooh Afza are widely used as nutritional supplements in the country. These plants and formulations, deeply ingrained in daily life, have been scientifically proven to possess immunomodulatory, antioxidant, and anti-infective properties. This scientific backing may contribute to the comparatively lower death rate among Indians per million of the population due to COVID-19, despite limited health infrastructure (61). Various spices commonly found in kitchens are combined as single or multiple agents, added to boiling water, and consumed throughout the day as a form of medication to treat COVID/flu-like symptoms. For example, Liquorice (*Glycyrrhiza glabra*), a well-known herb, contains glycyrrhizin, a compound that has shown higher efficacy than some common antiviral drugs in inhibiting the replication of the SARS virus by blocking the virus’s ability to penetrate host cells. Similarly, Ginger (*Zingiber officinale*), often consumed in aqueous or alcoholic extracts, has demonstrated anti-inflammatory effects, particularly in managing asthma, and has shown antiviral activity against respiratory viruses like HRSV (Human Respiratory Syncytial Virus), inhibiting viral attachment and internalisation. Glycyrrhizin, a key component in liquorice, demonstrated higher efficacy compared to common antivirals in inhibiting the replication of the SARS virus. It effectively hindered the virus’s adsorption and penetration processes (1). The aqueous and alcoholic extracts of ginger have shown promising anti-asthmatic effects by mitigating inflammation through the suppression of Th2-mediated immune responses. Additionally, the aqueous extract of ginger has demonstrated efficacy against HRSV-induced plaque formation on airway epithelium by inhibiting viral attachment and internalisation, with an IC50 value indicating its potency (61). *Allium sativum* (garlic) also exhibited antiviral properties against the avian influenza virus H9N2 in Vero cells, highlighting its potential protective effects (62). Giloy herb gained prominence following the onset of the COVID-19 pandemic due to its immunomodulatory and antiviral properties. The genome sequencing of Giloy has proven to be a breakthrough in controlling COVID-19 (63).

These herbs and traditional remedies have long been recognised for their therapeutic benefits, and their continued use during the COVID-19 pandemic highlights the cultural and historical importance of herbal medicine in India. However, it is crucial to consider Health Technology Assessment (HTA) when prescribing treatments or remedies to patients. HTA ensures that prescribed interventions are not only effective and safe but also economically viable and ethically sound. By incorporating HTA into clinical decision-making, healthcare providers can make well-informed choices that maximise patient benefits, minimise harm, and align with broader societal values. This approach promotes evidence-based care, ensuring that treatments are both scientifically proven and in the best interest of the patient while reflecting ethical and social considerations. Health Technology Assessment (HTA) is a multidisciplinary process that provides a comprehensive summary of the medical, social, economic, and ethical considerations surrounding the use of health technologies. The evaluation of health technologies inherently involves normative issues because the primary goal of healthcare technologies is to improve people’s lives—whether by enhancing health outcomes or alleviating suffering. This involves promoting well-being, which is a moral pursuit. Additionally, key HTA factors like safety and effectiveness raise ethical questions about how these concepts should be defined and measured, such as determining the acceptable thresholds for safety. Including ethics in HTA enhances its efficiency, ensuring that decisions are well-informed and aligned with societal and moral values, thus preventing conflicts between the outcomes of the assessment and the ethical principles held by society (64). Together, ethics committees and researchers have had to find ways to overcome these obstacles in order to ensure that clinical research continues to advance while protecting the rights and well-being of participants. This process requires collaboration, clear communication, and an ongoing commitment to upholding ethical standards in the face of an increasingly complex clinical research landscape (65).

While the use of home remedies and herbal treatments during the COVID-19 pandemic is deeply rooted in tradition and has shown some promising therapeutic properties, it is important to acknowledge the limitations and potential risks associated with this approach. Despite the scientific backing for some of these herbs, such as their immunomodulatory and anti-inflammatory effects, the efficacy of many home remedies in preventing or treating COVID-19 remains largely unproven in rigorous clinical trials. Moreover, reliance on home remedies can lead to delays in seeking appropriate medical care, especially in cases of severe illness, where timely intervention is critical. Additionally, without standardised dosages and proper medical supervision, the use of herbal treatments can pose safety risks, including potential interactions with prescribed medications or adverse side effects. Therefore, while these remedies may offer some benefits, it is crucial to approach them with caution and ensure that they are used as complementary treatments rather than substitutes for evidence-based medical care.

### 4.3 Limitations, strengths and recommendations

The primary limitation of this study is its generalisability, as the data may not reflect the entire population. This research represents the first comprehensive assessment of parental knowledge and attitudes regarding COVID-19 symptoms in children which considers various aspects, including modes of transmission, sources of information, protective measures, and parental satisfaction with government actions related to COVID-19 in India. This study aims to contribute valuable insights to the ongoing discourse surrounding public health awareness and parental engagement during these challenging periods.

Additionally, the investigation explored the associations between parents’ knowledge, attitudes, beliefs, and various sociodemographic variables. A reliable instrument was employed to assess knowledge and attitudes concerning COVID-19 in children. To enhance scientific validation, computational intelligence was utilised to analyse the data for specificity, sensitivity, accuracy, and precision.

Future studies are advised to further investigate the knowledge and attitudes surrounding COVID-19 symptoms, sources of infection, and modes of transmission across all age groups. Replication of this study would be beneficial to ensure external validity and generalizability. Furthermore, a comparative analysis between rural and urban populations could bring to the fore differences in knowledge, attitude, and practices (KAP) within these groups. Employing alternative research designs, beyond cross-sectional studies, could provide deeper insights into the adequate KAP regarding COVID-19 symptoms among children. Enhancing educational opportunities for parents would empower them to make informed decisions regarding preventive measures and timely medical intervention.

## 5 Conclusion

This research examines the cognitive dimensions of parental perspectives regarding COVID-19 in children, providing valuable insights into symptom recognition, transmission dynamics, and protective behaviours. The findings reveal that nearly 47.65% of surveyed parents are aged between 29 and 38, with 65.27% identifying as female, often accompanied by their mothers. Utilising GLM for analysis, the study indicates that habitation significantly impacts both knowledge levels and attitudes among parents. A significant majority (85%) of parents predominantly relied on news channels for information regarding COVID-19. In terms of their understanding of the virus, 52.9% exhibited good knowledge, while 43.2% demonstrated excellent knowledge. Notably, fever and cough were recognised by 95.6% of parents as the most identifiable symptoms in children, with 93.2% indicating that respiratory droplet infection is the primary route of transmission for the virus. Moreover, a substantial 94.7% of participants supported the decision to close schools, reflecting alignment with nationwide measures implemented in response to government directives aimed at curbing the spread of COVID-19. This study also highlights the performance of various machine learning classifiers, revealing that SVM classifiers achieved the highest accuracy of 66.70% with random sampling, while AB classifiers exhibited the lowest accuracy at 16.70%. By establishing correlations with sociodemographic parameters and employing advanced analytical techniques, the study enhances the depth of its analysis. Furthermore, the findings underscore the necessity for tailored educational programmes to address knowledge disparities, particularly in rural areas, across different genders, and varying socioeconomic statuses. The effectiveness of advanced analytics, as demonstrated by the SVM classifier, emphasises the potential for informed decision-making in public health communication and targeted interventions, ultimately empowering parents to safeguard the well-being of their children during the ongoing pandemic.

## Data Availability

The raw data supporting the conclusions of this article will be made available by the authors, without undue reservation.

## Footnotes

^1^ https://www.mygov.in/covid-19

^2^ https://www.bbc.com/news/world-asia-india-57551285

## References

[1] Tillu G Chaturvedi S Chopra A Patwardhan B. Public health approach of Ayurveda and yoga for COVID-19 prophylaxis. J Altern Complement Med. (2020) 26:360–4. doi: 10.1089/acm.2020.0129, PMID: 32310670

[2] S DP B KR K A Swamy S Bhodaji S Deshmukh A. Knowledge, attitude, awareness and practice towards Covid-19 pandemic in Indian citizens during the national lockdown period: A quick online cross-sectional study. Int J Physiother Res. (2020) 8:35043515. doi: 10.16965/ijpr.2020.139

[3] Lubrano R del Giudice E Marcellino A Ventriglia F Dilillo A de Luca E. Change in pediatric health care spending and drug utilization during the covid-19 pandemic. Child Aust. (2021) 8:1–12. doi: 10.3390/children8121183, PMID: 34943379 PMC8699860

[4] Kumar A Nayar KR Bhat LD. Debate: COVID-19 and children in India. Child Adolesc Mental Health. (2020) 25:165–6. doi: 10.1111/camh.12398, PMID: 32599669 PMC7361601

[5] Lee PI Hsueh PR. Emerging threats from zoonotic coronaviruses-from SARS and MERS to 2019-nCoV. J Microbiol Immunol Infect. (2020) 53:365–7. doi: 10.1016/j.jmii.2020.02.001, PMID: 32035811 PMC7102579

[6] Wang C Pan R Wan X Tan Y Xu L McIntyre RS. A longitudinal study on the mental health of general population during the COVID-19 epidemic in China. Brain Behav Immun. (2020) 87:40–8. doi: 10.1016/j.bbi.2020.04.028, PMID: 32298802 PMC7153528

[7] Ng CSM Ng SSL. Impact of the COVID-19 pandemic on children’s mental health: a systematic review. Front Psychol. (2022) 13:1–22. doi: 10.3389/fpsyt.2022.975936, PMID: 36329921 PMC9622998

[8] Breidokienė R Jusienė R Urbonas V Praninskienė R Girdzijauskienė S. Sedentary behavior among 6–14-year-old children during the COVID-19 lockdown and its relation to physical and mental health. Health. (2021) 9:1–14. doi: 10.3390/healthcare9060756, PMID: 34207421 PMC8235225

[9] Almeida IL d L Rego JF Teixeira ACG Moreira MR. Social isolation and its impact on child and adolescent development: a systematic review. Rev Paul Pediatr. (2022) 40:e2020385. doi: 10.1590/1984-0462/2022/40/2020385, PMID: 34614137 PMC8543788

[10] Liu Y Lu L Wang WX Liu S Chen HR Gao X. Job burnout and occupational stressors among chinese healthcare professionals at county-level health alliances. Int J Environ Res Public Health. (2020) 17:1–9. doi: 10.3390/ijerph17061848, PMID: 32178394 PMC7142970

[11] Ng CSM. Effects of workplace bullying on Chinese children’s health, behaviours and school adjustment via parenting: study protocol for a longitudinal study. BMC Public Health. (2019) 19:1–12. doi: 10.1186/s12889-019-6458-1, PMID: 30700297 PMC6354335

[12] Cheng TL Moon M Artman M. Shoring up the safety net for children in the COVID-19 pandemic. Pediatr Res. (2020) 88:349–51. doi: 10.1038/s41390-020-1071-7, PMID: 32712625

[13] Maguire A Ross E O’Reilly D. Parental mental health and risk of poor mental health and death by suicide in offspring: a population-wide data-linkage study. Epidemiol Psychiatr Sci. (2022) 31:e25. doi: 10.1017/S2045796022000063, PMID: 35438075 PMC9069591

[14] Fisher PA Beauchamp KG Roos LE Noll LK Flannery J Delker BC. The neurobiology of intervention and prevention in early adversity. Annu Rev Clin Psychol. (2016) 12:331–57. doi: 10.1146/annurev-clinpsy-032814-112855, PMID: 26666968

[15] Li J Huang C Yang Y Liu J Lin X Pan J. How nursing students’ risk perception affected their professional commitment during the COVID-19 pandemic: the mediating effects of negative emotions and moderating effects of psychological capital. Humanit Soc Sci Commun. (2023) 10:195–9. doi: 10.1057/s41599-023-01719-6, PMID: 37192948 PMC10156579

[16] Ding X Wang L Sun J Li DY Zheng BY He SW. Effectiveness of empathy clinical education for children’s nursing students: a quasi-experimental study. Nurse Educ Today. (2020) 85:104260. doi: 10.1016/j.nedt.2019.104260, PMID: 31778862

[17] Abuhammad S. Parents’ knowledge and attitude towards COVID-19 in children: a Jordanian study. Int J Clin Pract. (2021) 75:e13671–6. doi: 10.1111/ijcp.13671, PMID: 32780560 PMC7435566

[18] Gottlieb M Bridwell R Ravera J Long B. Multisystem inflammatory syndrome in children with COVID-19. Am J Emerg Med. (2021) 49:148–52. doi: 10.1016/j.ajem.2021.05.076, PMID: 34116467 PMC8185530

[19] Dufort EM Koumans EH Chow EJ Rosenthal EM Muse A Rowlands J. Multisystem inflammatory syndrome in children in New York state. N Engl J Med. (2020) 383:347–58. doi: 10.1056/nejmoa2021756, PMID: 32598830 PMC7346766

[20] Ahmad A Khan MU Patel IB Bandari DK. 116th annual meeting of the American Association of Colleges of Pharmacy National Harbor, Maryland, 2015. Am J Pharm Educ. (2015) 79:S4. doi: 10.5688/ajpe795S4

[21] Person B Sy F Holton K Govert B Liang A Garza B. Fear and stigma: the epidemic within the SARS outbreak. Emerg Infect Dis. (2004) 10:358–63. doi: 10.3201/eid1002.030750, PMID: 15030713 PMC3322940

[22] Zhong BL Luo W Li HM Zhang QQ Liu XG Li WT. Knowledge, attitudes, and practices towards COVID-19 among chinese residents during the rapid rise period of the COVID-19 outbreak: a quick online cross-sectional survey. Int J Biol Sci. (2020) 16:1745–52. doi: 10.7150/ijbs.45221, PMID: 32226294 PMC7098034

[23] Idele P Anthony D You D Luo C Mofenson L. The evolving picture of SARS-CoV-2 and COVID-19 in children: critical knowledge gaps. BMJ Glob Health. (2020) 5:e003454. doi: 10.1136/BMJGH-2020-003454, PMID: 32938610 PMC7496567

[24] Banerjee S Guha A Das A Nandi M Mondal R. A preliminary report of COVID-19 in children in India. Indian Pediatr. (2020) 57:963–4. doi: 10.1007/s13312-020-2004-6, PMID: 32729849 PMC7605489

[25] Sun J Xu Y Qu Q Luo W. Knowledge of and attitudes toward COVID-19 among parents of child dental patients during the outbreak. Braz Oral Res. (2020) 34:1–8. doi: 10.1590/1807-3107BOR-2020.VOL34.0066 32520076

[26] Sumbul Sultana A Heyat MBB Rahman K Akhtar F Parveen S. Efficacy and classification of Sesamum indicum Linn seeds with Rosa damascena mill oil in uncomplicated pelvic inflammatory disease using machine learning. Front Chem. (2024) 12:1–21. doi: 10.3389/fchem.2024.1361980, PMID: 38629105 PMC11018920

[27] Fazmiya MJA Sultana A Heyat MBB Parveen S Rahman K Akhtar F. Efficacy of a vaginal suppository formulation prepared with *Acacia arabica* (lam.) Willd. Gum and *Cinnamomum camphora* (L.) J. Presl. In heavy menstrual bleeding analyzed using a machine learning technique. Front Pharmacol. (2024) 15:1–23. doi: 10.3389/fphar.2024.1331622, PMID: 38410133 PMC10894987

[28] Sultana A Akhtar F Bin Heyat MB Singh P Parveen S Kumar S. “Unveiling the efficacy of Unani medicine in female disorders through machine learning: current challenges and opportunities,” In *2023 20th international computer conference on wavelet active media technology and information processing, ICCWAMTIP 2023, IEEE*, (2023), pp. 1–6.

[29] Saleem SM Jan SS. Modified Kuppuswamy socioeconomic scale updated for the year 2021. Indian J Forensic Community Med. (2021) 8:1–3. doi: 10.18231/j.ijfcm.2021.001

[30] Chowdhury S Chakraborty PP. Universal health coverage - there is more to it than meets the eye. J Family Med Prim Care. (2017) 6:169–70. doi: 10.4103/jfmpc.jfmpc 29026777 PMC5629889

[31] Sultana A Begum W Saeedi R Rahman K Bin Heyat MB Akhtar F. Experimental and computational approaches for the classification and correlation of temperament (Mizaj) and uterine Dystemperament (Su’-I-Mizaj Al-Rahim) in abnormal vaginal discharge (Sayalan Al-Rahim) based on clinical analysis using support vector machine. Complexity. (2022) 2022:1–16. doi: 10.1155/2022/5718501

[32] Bin Heyat MB Akhtar F Abbas SJ al-Sarem M Alqarafi A Stalin A. Wearable flexible electronics based cardiac electrode for researcher mental stress detection system using machine learning models on single Lead electrocardiogram signal. Bios. (2022) 12:427. doi: 10.3390/bios12060427, PMID: 35735574 PMC9221208

[33] Ullah H Heyat MBB Akhtar F Muaad AY Ukwuoma CC Bilal M. An automatic premature ventricular contraction recognition system based on imbalanced dataset and pre-trained residual network using transfer learning on ECG signal. Diagnostics. (2023) 13:87. doi: 10.3390/diagnostics13010087, PMID: 36611379 PMC9818233

[34] Heyat MBB Akhtar F Munir F Sultana A Muaad AY Gul I. Unravelling the complexities of depression with medical intelligence: exploring the interplay of genetics, hormones, and brain function. Complex Intell Syst. (2024) 10:5883–915. doi: 10.1007/s40747-024-01346-x

[35] Benifa JVB Chola C Muaad AY Hayat MAB Bin Heyat MB Mehrotra R. FMDNet: an efficient system for face mask detection based on lightweight model during COVID-19 pandemic in public areas. Sensors. (2023) 23:6090. doi: 10.3390/s23136090, PMID: 37447939 PMC10346139

[36] Akhtar F Heyat MBB Parveen S Singh P Hassan MFU Parveen S. “Early coronary heart disease deciphered via support vector machines: insights from experiments,” In *2023 20th international computer conference on wavelet active media technology and information processing, ICCWAMTIP 2023, IEEE*, (2023), pp. 1–7. doi: 10.1109/ICCWAMTIP60502.2023.10387051

[37] Parveen S Bin Heyat MB Akhtar F Parveen S Asrafali B Singh B. “Interweaving artificial intelligence and bio-signals in mental fatigue: unveiling dynamics and future pathways,” In *2023 20th international computer conference on wavelet active media technology and information processing, ICCWAMTIP 2023, IEEE*, (2023), pp. 1–9.

[38] Vabalas A Gowen E Poliakoff E Casson AJ. Machine learning algorithm validation with a limited sample size. PLoS One. (2019) 14:e0224365. doi: 10.1371/journal.pone.0224365, PMID: 31697686 PMC6837442

[39] Varoquaux G. Cross-validation failure: small sample sizes lead to large error bars. NeuroImage. (2018) 180:68–77. doi: 10.1016/j.neuroimage.2017.06.061, PMID: 28655633

[40] Sari FR Suwarsono EA Adhiyanto C Habibi AA Siregar AS Ariany D. Epidemiological assessment of COVID-19 clinical symptoms and its associated factors from Banten districts: the role of gender aspects. Bangladesh J Med Sci. (2022) 21:782–7. doi: 10.3329/bjms.v21i4.60248

[41] Diaferio L Parisi GF Brindisi G Indolfi C Marchese G Ghiglioni DG. Cross-sectional survey on impact of paediatric COVID-19 among Italian paediatricians: report from the SIAIP rhino-sinusitis and conjunctivitis committee. Ital J Pediatr. (2020) 46:146–6. doi: 10.1186/s13052-020-00906-4, PMID: 33023616 PMC7538039

[42] Chen N Zhou M Dong X Qu J Gong F Han Y. Epidemiological and clinical characteristics of 99 cases of 2019 novel coronavirus pneumonia in Wuhan, China: a descriptive study. Lancet. (2020) 395:507–13. doi: 10.1016/S0140-6736(20)30211-7, PMID: 32007143 PMC7135076

[43] Huang C Wang Y Li X Ren L Zhao J Hu Y. Clinical features of patients infected with 2019 novel coronavirus in Wuhan, China. Lancet. (2020) 395:497–506. doi: 10.1016/S0140-6736(20)30183-5, PMID: 31986264 PMC7159299

[44] Newell R El-Shakankery K Bhowmik A Rajakulasingam RK. Panton-valentine leucocidin *Staphylococcus aureus* necrotising pneumonia in a clinically well patient. Br J Hosp Med. (2023) 84:1–4. doi: 10.12968/hmed.2022.0396 36989153

[45] Jiang R. Diagnosis, treatment and prevention of 2019 novel coronavirus infection in children: experts’ consensus statement (third edition). Chinese J Appl Clin Pediatr. (2021) 36:721–32. doi: 10.3760/cma.j.cn101070-20210226-00235 PMC7090771 32034659

[46] Sinha IP Harwood R Semple MG Hawcutt DB Thursfield R Narayan O. COVID-19 infection in children. Lancet Respir Med. (2020) 8:446–7. doi: 10.1016/S2213-2600(20)30152-1, PMID: 32224304 PMC7154504

[47] Adalja AA Toner E Inglesby TV. Priorities for the US health community responding to COVID-19. JAMA - J Am Med Assoc. (2020) 323:1343–4. doi: 10.1001/jama.2020.3413 32125355

[48] Chinazzi M Davis JT Ajelli M Gioannini C Litvinova M Merler S. The effect of travel restrictions on the spread of the 2019 novel coronavirus (COVID-19) outbreak. Science. (2020) 368:395–400. doi: 10.1126/science.aba9757, PMID: 32144116 PMC7164386

[49] Wu Z McGoogan JM. Characteristics of and important lessons from the coronavirus disease 2019 (COVID-19) outbreak in China: summary of a report of 72314 cases from the Chinese Center for Disease Control and Prevention. JAMA - J Am Med Assoc. (2020) 323:1239–42. doi: 10.1001/jama.2020.2648, PMID: 32091533

[50] Aman F Masood S. How nutrition can help to fight against covid-19 pandemic. Pakistan J Med Sci. (2020) 36:S121–3. doi: 10.12669/pjms.36.COVID19-S4.2776, PMID: 32582329 PMC7306972

[51] Jefferson T. Interventions for the interruption or reduction of the spread of respiratory viruses. Cochrane Database Syst Rev. (2007) 4. doi: 10.1002/14651858.CD006207.pub2 17943895

[52] Schlapbach LJ Straney L Gelbart B Alexander J Franklin D Beca J. Burden of disease and change in practice in critically ill infants with bronchiolitis. Eur Respir J. (2017) 49:1601648. doi: 10.1183/13993003.01648-2016, PMID: 28572120

[53] Lubrano R Bloise S Testa A Marcellino A Dilillo A Mallardo S. Assessment of respiratory function in infants and young children wearing face masks during the COVID-19 pandemic. JAMA Netw Open. (2021) 4:E210414. doi: 10.1001/jamanetworkopen.2021.0414, PMID: 33651109 PMC7926283

[54] Antonova EN Rycroft CE Ambrose CS Heikkinen T Principi N. Burden of paediatric influenza in Western Europe: a systematic review. BMC Public Health. (2012) 12:1. doi: 10.1186/1471-2458-12-968, PMID: 23146107 PMC3534559

[55] Esposito S Principi N. To mask or not to mask children to overcome COVID-19. Eur J Pediatr. (2020) 179:1267–70. doi: 10.1007/s00431-020-03674-9, PMID: 32388722 PMC7210459

[56] Manisalidis I Stavropoulou E Stavropoulos A Bezirtzoglou E. Environmental and health impacts of air pollution: a review. Front Public Health. (2020) 8:1–13. doi: 10.3389/fpubh.2020.00014, PMID: 32154200 PMC7044178

[57] Brendish NJ Poole S Naidu VV Mansbridge CT Norton NJ Wheeler H. Clinical impact of molecular point-of-care testing for suspected COVID-19 in hospital (COV-19POC): a prospective, interventional, non-randomised, controlled study. Lancet Respir Med. (2020) 8:1192–200. doi: 10.1016/S2213-2600(20)30454-9, PMID: 33038974 PMC7544498

[58] Fegert JM Vitiello B Plener PL Clemens V. Challenges and burden of the coronavirus 2019 (COVID-19) pandemic for child and adolescent mental health: a narrative review to highlight clinical and research needs in the acute phase and the long return to normality. Child Adolesc Psychiatry Ment Health. (2020) 14:11–20. doi: 10.1186/s13034-020-00329-3, PMID: 32419840 PMC7216870

[59] Borse P. B. N. Vivek*, Konwar Aditya Narayan, “Since January 2020 Elsevier has created a COVID-19 resource Centre with free information in English and mandarin on the novel coronavirus COVID-19. The COVID-19 resource Centre is hosted on Elsevier connect, the company’s public news and information,” Psychiatry Res, vol. 14:293, (2020).

[60] Hu F Ma Q Hu H Zhou KH Wei S. A study of the spatial network structure of ethnic regions in Northwest China based on multiple factor flows in the context of COVID-19: evidence from Ningxia. Heliyon. (2024) 10:e24653. doi: 10.1016/j.heliyon.2024.e24653, PMID: 38312651 PMC10835267

[61] Ahmad S Zahiruddin S Parveen B Basist P Parveen A Gaurav. Indian medicinal plants and formulations and their potential against COVID-19–preclinical and clinical research. Front Pharmacol. (2021) 11:1–34. doi: 10.3389/fphar.2020.578970, PMID: 33737875 PMC7962606

[62] Rasool A Khan MU Ali MA Anjum AA Ahmed I Aslam A. Anti-avian influenza virus H9N2 activity of aqueous extracts of Zingiber officinalis (ginger) & *Allium sativum* (garlic) in chick embryos. Pak J Pharm Sci. (2017) 30:1341–4. PMID: 29039335

[63] Arora S Goyal A Rawat DS Samantha K. Giloy: a potential anti-COVID-19 herb with propitious pharmacological attributes: a short review. J Biomol Struct Dyn. (2023) 41:7001–8. doi: 10.1080/07391102.2022.2110157, PMID: 35950530

[64] Lysdahl KB Oortwijn W van der Wilt G Refolo P Sacchini D Mozygemba K. Ethical analysis in HTA of complex health interventions. BMC Med Ethics. (2016) 17:1–15. doi: 10.1186/s12910-016-0099-z, PMID: 27004792 PMC4804607

[65] Minacori R Refolo P Sacchini D Spagnolo AG. Research ethics committees and clinical research in Italy: where are we going? Eur Rev Med Pharmacol Sci. (2015) 19:481–5. PMID: 25720722

